# Genetic diversity of *Anadara tuberculosa* in two localities of the Colombian Pacific Coast

**DOI:** 10.1038/s41598-024-78869-3

**Published:** 2024-11-18

**Authors:** Luis Fuentes, Marcela Guevara-Suarez, María Mercedes Zambrano, Pedro Jiménez, Jorge Duitama, Silvia Restrepo

**Affiliations:** 1 Laboratory of Mycology and Phytopathology (LAMFU), Department of Biological Sciences, Department of Food and Chemical Engineering, Universidad de Los Andes, Bogotá, Colombia; 2 Applied genomics research group, Vice president of Research, Universidad de Los Andes, Bogotá, Colombia; 3 Corpogen Research Center, Bogotá, Colombia; 4 Faculty of Basic and Applied Sciences, Universidad Militar Nueva Granada, Cajicá, Colombia; 5 Department of System and Computing Engineering, Universidad de Los Andes, Bogotá, Colombia; 6 Boyce Thompson Institute, Ithaca, NY USA

**Keywords:** Computational biology and bioinformatics, Ecology, Genetics, Molecular biology

## Abstract

Piangua, *Anadara tuberculosa*, is an economically important mollusk for the human population living on the Colombian Pacific Coast. In the last years, the demand and exploitation of this mollusk have increased, putting it at risk to the point of being endangered. This research aimed to identify the genetic diversity and population structure of piangua in two localities on the Pacific Coast of Colombia. We assembled a chromosome-level genome using PacBio-Hifi and Arima sequencing. We obtained 274 scaffolds with an N50 of 45.42 Mbp, a total size of 953 Mbp, and a completeness of 91% based on BUSCO scores. The transposable elements accounted for 30.29% of the genome, and 24,317 genes were annotated. Genome-guided variant calling for 89 samples using DArT sequencing data delivered 4,825 bi-allelic SNPs, which supported genetic diversity and population structure analyses. Data showed that the piangua populations in the two localities were under expansion events more than 100k years ago. However, results also showed a reduction in genetic diversity, as evidenced by the loss of heterozygosity, which may be caused by high levels of inbreeding, probably due to a recent overexploitation. Furthermore, although we evidenced gene flow between the two localities, there is also a subtle geographical population structure between the two localities and among mangroves in one of the localities. This is the first study in Colombia that provides relevant genetic information on piangua to lay the foundations for conservation strategies.

**Supplementary Information:**

The online version contains supplementary material available at 10.1038/s41598-024-78869-3.

## Introduction

Colombia is considered one of the most biodiverse countries in the world, with approximately 10% of the diversity of the Earth^1^, and one of the Colombian most diverse regions is the Pacific coast. This region belongs to the Chocó biogeographic region, considered the ninth most biodiverse hotspot in the world^2–4^. However, social indicators on the Colombian Pacific coast are among the worst in the country^2,5^. Consequently, the communities have seen the need to exploit natural resources as economic and nutritional sources through mining, hunting, and fishing^2^.

On the Colombian Pacific coast, mollusk fishing is carried on in the mangroves, an ecosystem rich in biodiversity^6^. In the last years, shellfish extraction has increased as it has become an important economic activity for local communities, mainly in regions such as Bahía Málaga, Bahía Solano, Iscuandé, and Tumaco^7^, where the piangua (*Anadara tuberculosa*) is one of the mollusks with significant importance. Demand for the piangua has led to high capture levels in Colombia, where its export increased from 100 tons/year to 3,283 tons/year between 1980 and 2004^8^. This led to a reduction of piangua of up to 60% in Bahía Málaga^9^, while in Tumaco, the per capita fishing rate has decreased considerably since 1975^5^. This effect is also reported for Ecuador and Perú^1^.

Deforestation, overfishing, and overharvesting are the most important threats to biodiversity of marine species in Colombia. These phenomena generate important genetic losses between and within species, evidenced by a decrease in effective population size, which induces inbreeding and accelerates the reduction of diversity through genetic drift. This process leads to a reduction in species fitness, which could lead to extinction.

The decrease in the piangua population has placed this mollusk among the endangered species^10^. For all these reasons, the Colombian government is committed to adopting bioeconomy (Global Bioeconomy Summit Communiqué, 2018) as a sustainable approach to exploiting its natural resources. Within this framework, scientific knowledge of the diversity and population genomics of piangua is crucial because it will provide information for the pursuit of conservation strategies and sustainable exploitation.

Population genetics studies in different species use reduced representation DNA sequencing (RRS) approaches, such as DArTseq^11^. Although the analysis of RRS data can be performed *de novo*^12^, using a reference genome can lead to more robust information to infer population parameters and diversity trends^13^. The *de novo* assembly of eucaryote genomes remains challenging due to the quality and length of DNA sequencing reads needed to assemble genomes with a high percentage of repetitive sequences and high heterozygosity^14^. High-quality chromosome-level assemblies for some complex genomes have been achieved using a combination of long-read sequencing, such as PacBio or NanoPore, and scaffolding techniques, such as the high-throughput chromatin conformation capture (Hi-C) approach^15,16^. The hybrid methodology has allowed the chromosome-level assembly of *Anadara brougthonii* and *Anadara kagoshimensis*, two species related to *A. tuberculosa*^17,18^.

**Fig. 1:**
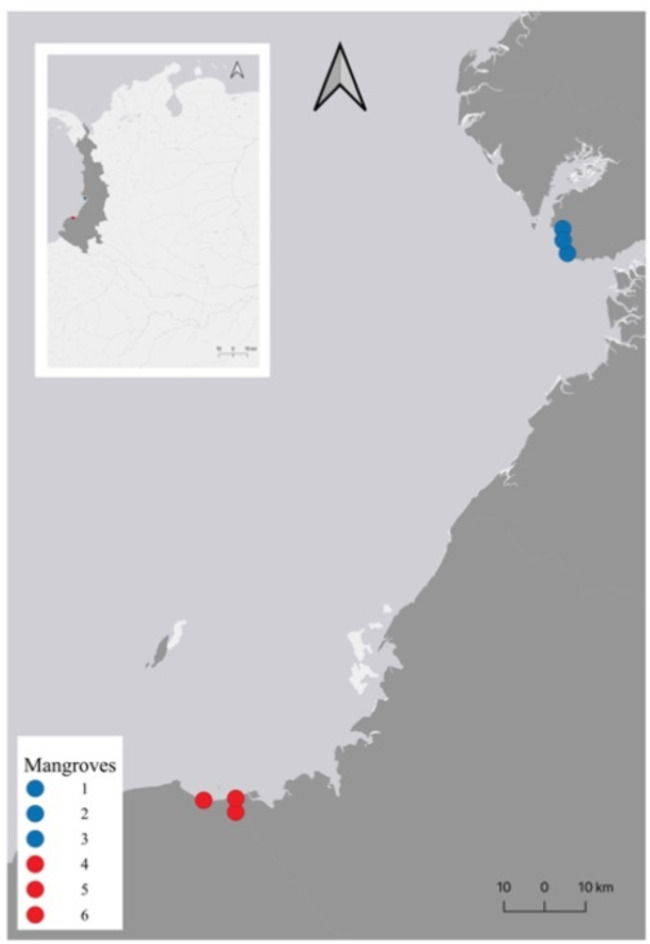
Map of sampling points. The Pacific region is represented in dark-gray color. The sampling points (mangroves) from Buenaventura are in blue, and the sampling points from Iscuandé are in red. The map was made using QGIS (https://www.qgis.org/es/site/).

Therefore, in this study, we report a chromosome-level genome assembly of *A. tuberculosa*, and its use as a reference in the analysis of its genetic diversity and population structure in two regions of the Colombian Pacific coast. Despite our initial goal to sample along the complete Pacific coast of Colombia, our sampling was limited by socioeconomic and political limitations, making it difficult to fully cover the species’ range. Although we limited our analysis and conclusions to two localities (Fig. 1), we gathered important information to unravel the genetic situation of the species in this understudied region. The results are relevant for discussing conservation strategies with the appropriate stakeholders.

## Results

### The genome of *Anadara tuberculosa*

We assembled a genome combining Pac-Bio-Hifi and Hi-C sequencing data. A total of 33 Gb of Pac-Bio Hifi data, containing 3,698,171 raw reads with a mean length of 12,751 base pairs and a quality > 34, were used for the assembly. The primary assembly obtained running the Hifiasm software had a size of 953 Mbp, fragmented into 366 contigs with an N50 of 9.96 Mbp and a 28X median read depth. Scaffolding of these contigs was performed using 77 Gb of Hi-C pair-end sequencing data (Table 1). A total of 525,668,541 reads were aligned to the primary assembly of Hifiasm with a 78.31% overall alignment rate. Scaffolding was performed using this alignment, obtaining 274 scaffolds with a scaffold N50 of 45.419 Mbp. The mitochondrial genome was identified as scaffold 212 of assembly with a size of 38 Kbp. It showed 80.3% identity with the mitochondrial genome of *Anadara kagoshimensis* and 71.5% identity with that of *Anadara brougthonii.*

**Table 1:** Summary statistic for the Chromosome-level genome.

PacBio - Hifi	Reads	3,698,171
	Contigs	366
	N50	9.93 Mbp
	L50	30
	GC	32.39%
	Length	953.1 Mbp
	Depth	28X
Arima	Reads	525,668,541
	Scaffolds	274
	N50	45.42 Mbp
	L50	9
	GC	32.39%
	Length	953.1 Mbp
	Depth	28X
BUSCO	Single-copy	87.6%
	Duplicated	3.4%
	Fragmented	2.8%
	Missing	6.2%

**Fig. 2:**
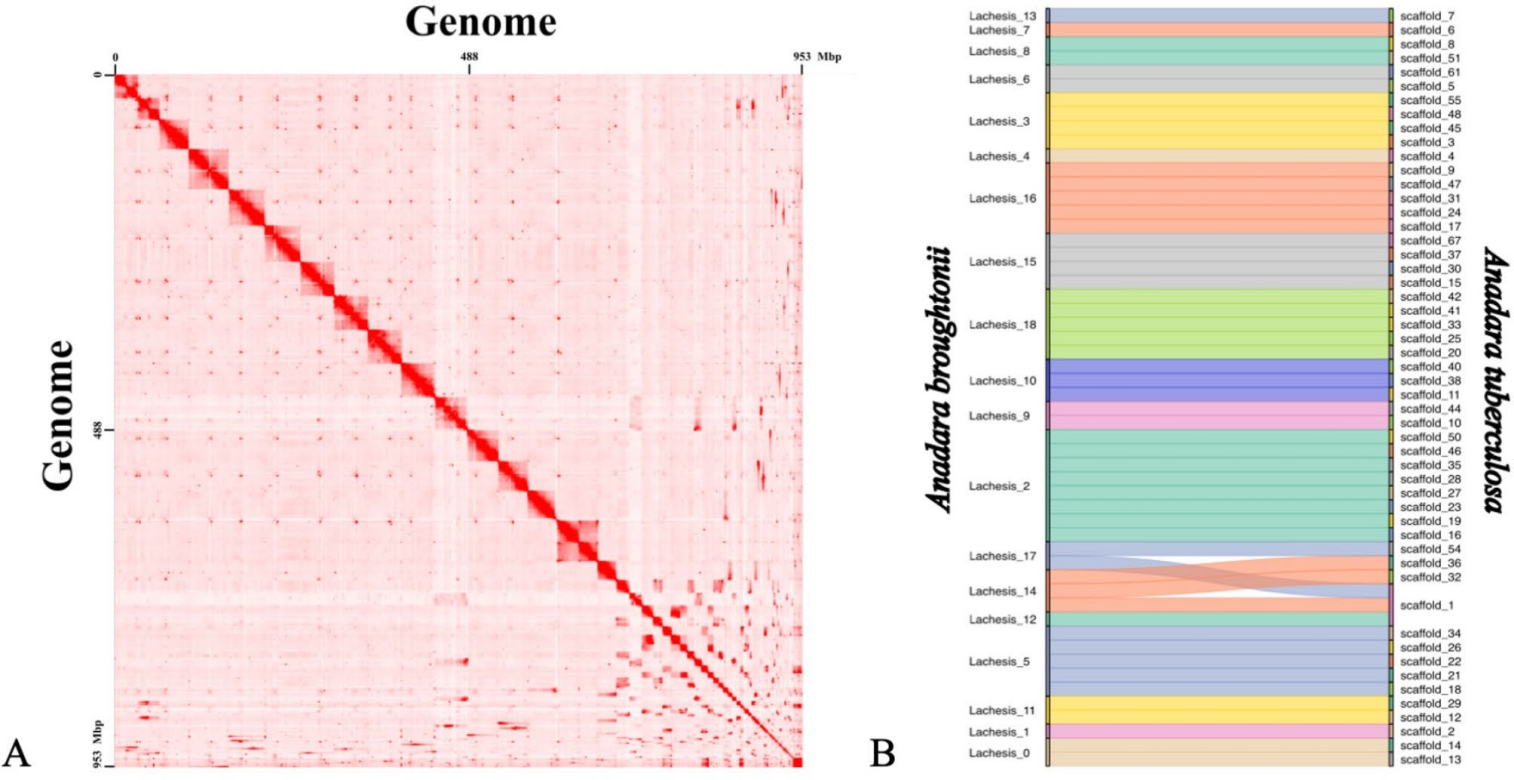
Genomic analysis. (**A**) Hi-C heatmap. The x-axis and y-axis represent the 274 scaffolds. Intense-red dots show the interactions between regions. (**B**) Synteny diagram between the chromosome-level genome of *A. tuberculosa* and the chromosome-level genome of *A. broughtonii*.

Based on the alignment of conserved genes in the Mollusca phylum, this primary assembly had a predicted completeness of 91% [Single-Copy: 87.6%, Duplicated: 3.4%], with 2.8% of fragmented genes and 6.2% of missing genes. According to the Hi-C heat map (Fig. 2A), the intense red diagonal indicates that the scaffolds showed a well-organized intra-chromosomal interaction. However, the intense red dots away from the diagonal indicate many interactions between scaffolds, mainly between small scaffolds.

.

We annotated transposable elements (TEs), accounting for 30.29% of the genome. The DNA transposons spanned 20.66%, and the LTR elements spanned 8.86%. After masking regions with TE annotations, a total of 24,305 genes were annotated with an average CDS and protein length of 1,264.79 bp and 420.45 bp, respectively. A synteny analysis of orthologous genes between the annotated genome assemblies of *A. tuberculosa* and *A. broughtonni* revealed 511 blocks, from which 52 *A. tuberculosa* scaffolds shared homologous genes with the 19 *A. broughtonni* chromosomes (Fig. 2B). From these, the largest scaffold (scaffold one) was paired with the chromosomes 12, 14, and 17 of *A. broughtonni* (crossed lines in Fig. 2A).

For the functional analysis (Fig. S1), only 18,760 of the 24,305 predicted proteins were classified, representing 92.4%. These proteins were characterized in Clusters of Orthologous Groups (COGs), which, in turn, were classified into four general categories. Thus, 37.9% of proteins were classified in the cellular processes and signaling category, 18.6% in metabolism, 17% in information storage and processing, and 26.4% in poorly characterized. The proteins present in the last category were proteins of unknown function.

### Identification of the SNPs of the* A. tuberculosa* populations

Genotyping was performed by mapping the DArTseq reads to the chromosome-level genome presented above. An average of 2,000,000 trimmed and filtered reads for each sample were mapped with an overall alignment rate of 85%. A total of 78,889 SNPs were initially identified. After applying filters, the number of bi-allelic SNPs was reduced to 4,825, with a missing data of 21.5%. Out of all these SNPs, 1,381 rejected the assumptions of Hardy-Weinberg Equilibrium (HWE) with a corrected p-value < 0.01. The minor allele frequency (MAF) distribution (Fig. S2) showed that 3,293 SNPs had frequencies lower than (0.1) A total of 356 of these SNPs belonged to the 1,381 HWE-rejected SNPs. Moreover, 49% of HWE-rejected SNPs had frequencies lower than (0.2) To compare diversity information among localities, the samples were classified by localities, and population statistics were recalculated keeping all 4,825 SNPs within each locality.

### Subtle genetic structure of piangua populations in two localities on the Pacific coast

Genetic diversity in both sampling points was similar, as assessed by the similarity of MAF distributions (Fig. S3). More than half of the SNPs had minor allele frequencies below < 0.1. Specifically, the minor alleles of 826 SNPs were segregating only in Buenaventura, while minor alleles of 788 SNPs were segregating only in Iscuandé (Fig. S8). These private SNPs represent approximately 16.6% of all SNPs. In 97% of the cases, the minor allele frequency of these SNPs within their population was lower than 0.1. Consequently, only 16 SNPs from Buenaventura and 27 SNPs from Iscuandé rejected the HWE assumption.

**Fig. 3:**
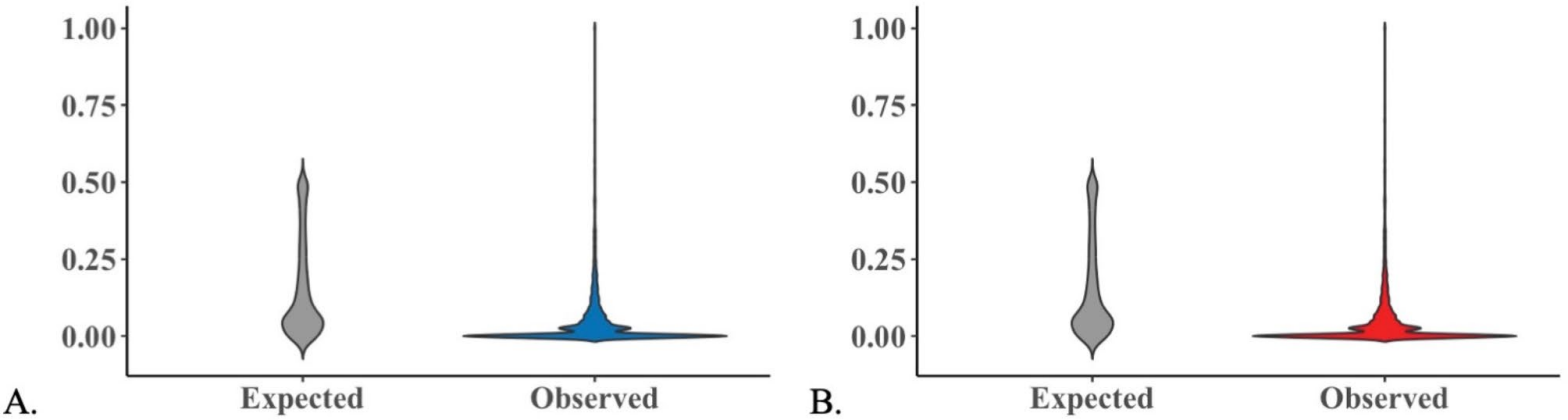
Violin plot of the distribution of the heterozygosity in both localities: (**A**) Heterozygosity density distribution for Buenaventura, the gray violin representing the expected heterozygosity density, and the blue violin representing the observed heterozygosity density. (**B**) Heterozygosity density distribution for Iscuandé. The gray violin representing the expected heterozygosity density, and the red violin representing the observed heterozygosity density. The x-axis is the frequency of heterozygosity.

A loss of heterozygosity was evident due to a high difference between expected (H_e_) and observed (H_o_) heterozygosity in both regions. In Buenaventura, the average H_e_ was 0.154 while the average H_o_ was 0.048, indicating a loss of heterozygosity of about 68.8%. Similarly, in Iscuandé, the average H_e_ was 0.166 while the average H_o_ was 0.045, indicating a loss of heterozygosity of about 72.9%. The distribution of heterozygosity was similar in both regions, where ~ 86% of SNPs had frequencies below 0.1 (Fig. 3).

Nucleotide diversity was compared between both localities, 5.57e^− 8^ in Buenaventura and 6.03e^− 8^ in Iscuandé. This showed no difference in nucleotide diversity between them and corroborated the MAF distribution results. The fixation index, F_st_, showed low differentiation in the allele frequencies among the two localities (0.004) (Table 2). The low differentiation was constant within each locality because the pairwise F_st_ between mangrove swamps did not exceed 0.0104, and the Fst values between Iscuandé sampling points were negative (Table S1). Although the highest pairwise F_st_ was 0.01, some F_st_ values were significantly higher than zero, suggesting an incipient population differentiation. Two of these cases were observed between sampling points within Buenaventura. The inbreeding coefficient (F_is_) and the total inbreeding coefficient (F_it_) were high when the samples were not filtered by locality, with F_is_ of 0.713 and F_it_ of 0.714. However, these nonrandom mating indexes were also high within localities, 0.655 and 0.656 in Buenaventura and 0.684 in Iscuandé for both indexes (Table 2). This suggests that the variation within localities can explain most of the total variation.

**Table 2:** Population genetics values of piangua populations.

Locality	Buenaventura		Iscuandé
Samples	49		40
F_st_	0.004		
F_is_	0.713*		
	0.655		0.684
F_it_	0.714*		
	0.656		0.684
H_e_	0.155		0.166
H_o_	0.048		0.045
π	5.57e-^08^		6.03e-^08^
Tajima’ D	-0.11		-0.10

**Fig. 4:**
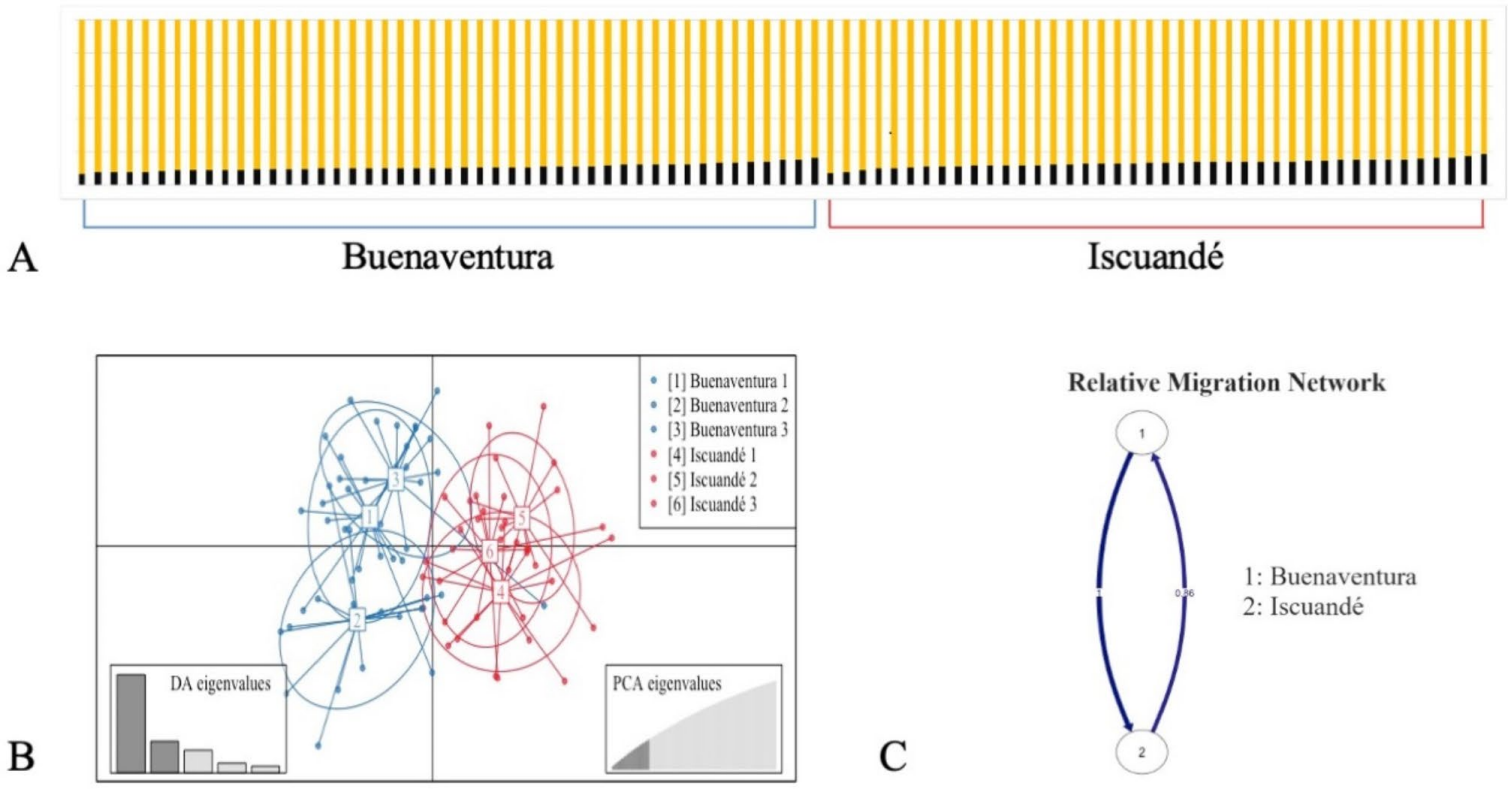
Population genetic analysis. (**A**) Cluster distribution by sample, according to Evanno et al. method. Thus, each cluster was represented by one color (black or yellow), and each bar corresponds to a sample. A total of 49 samples from Buenaventura (left) and 40 samples from Iscuandé (right) were sorted by the proportion of one cluster. Each bar represents an individual and colors are the genetic cluster of each individual. (**B**) The DAPC cross-validation. The clusters 1–3 represented the Buenaventura sampling points and the clusters 4–6 represented the Iscuandé sampling points. (**C**) Guided graph representing gene flow between the Buenaventura (1) and Iscuandé (2) samples. Blue arrows indicate genetic migration between localities.

According to the population genetic analysis (Fig. 4), there is a subtle genetic differentiation based on the geographical origin of the specimens. The STRUCTURE analysis (Fig. 4A) showed that neither locality was represented by a single cluster (yellow or black); however, admixture was evident between both localities. Although the Evanno method suggested that the best clustering for the data was two (Fig. S4A), the raw likelihood does not increase with the number of possible populations (Fig. S4B). The result of the LEA (Fig. S4C) and the NetView (Fig. S5) tests corroborated the admixture shown by STRUCTURE. However, the DAPC analysis (Fig. 4B) resolved partially overlapping clusters showing a subtle, limited genetic structure between the two sampled localities.

The admixture effect found in STRUCTURE was also evident when the specimens were sorted by mangrove (sampling point) to assess if there was a structure within localities. The gene flow analysis showed comparable gene exchange at all sampling points, with a minimum flow of 6% from mangrove five to mangrove three (Fig. S6), and with a mean of 93% between locations (Fig. 4C).

### Demography history

The extended Bayesian skyline plot (EBSP) revealed two significant population expansion events, the first around 130K years ago and the second around 100K years ago followed by a period of population stability (Fig. S7). This finding was supported by the neutrality test, where the Tajima’ D index yielded negative results for both localities, with − 0.11 for Buenaventura and − 0.10 for Iscuandé (Table 2). However, recent trends indicate a rapid population decline, as evidenced by the confidence intervals (CIs) for effective population size (Ne) where the values for Buenaventura, Iscuandé and the combined population fall below one thousand (Table S2).

## Discussion

Human impact is one of the main reasons for the loss of biodiversity^19,20^. Hence, it has been necessary to propose conservation strategies that consider information on genetic variability between and within species^21^. In recent years, genetic information has increased with the development of high-throughput sequencing technologies that have improved genetic knowledge in marine species^22–24^. Conservation strategies have also been proposed based on monitoring epigenetic markers and performing transcriptome assays^25^. In *A. tuberculosa*, the conservation strategies have been deficient, making it necessary to improve genetic knowledge of this mollusk. In this manuscript, we present the results of our efforts to use high throughput sequencing (HTS) through reduced representation libraries to assess the genetic diversity and population structure of *A. tuberculosa* in two localities on the Pacific Coast of Colombia. The samplings analyzed in this study represent only a subset of the full distribution of Piangua along the Colombia Pacific coast. It is important to acknowledge that the inference of the genetic values and population numbers inferred from this study are geographically limited due to the incomplete samples caused by the constraints mentioned in the introduction^26,27^. However, this is the first study using a technique based on HTS to obtain information on genetic diversity in Piangua, and the analysis will allow us to plan suitable conservation strategies with the local communities. We also offer the first chromosome-level genome for this species, an important resource for future genetic studies.

### Piangua genome

The lack of genetic information on Piangua could limit understanding of its biological and ecological processes. A genome of high quality and contiguity plays a crucial role in obtaining the best representation of the genetic diversity of species and understanding SNP distribution throughout the genome^28^. The chromosome-level genome reported in this study is the first genome of Piangua species and is of great relevance in subsequent studies.

We reported a genome of 953.1 Mbp, with an N50 scaffold of 45.42 Mbp and 274 scaffolds. These statistics were close to the genomes reported in *S. kagoshimensis*^18^ and *A. broughtonii*^17^, two species of the same genus. Using synteny analysis, we observed that the genome was at the chromosomal level, as our scaffolds reached completeness for all chromosomes reported in the *A. brougthonni* genome (Fig. 2). Furthermore, our genome at the chromosomal level had a completeness comparable to *Crassostrea gigas* (95.6%), which is the most investigated molluscan genome^29^, and with related species.

In terms of annotation, we found that 30.29% of the genome is represented by TEs. Recent studies have shown the importance of TEs as drivers of phenotypic variation in eukaryotes^30–32^. Within these regions, 8.86% of the genome corresponds to long terminal repeats (LTRs). LTRs are spread out throughout the Mollusca phylum, and they provide information about the genome evolution processes in this phylum^31^. Furthermore, the number of TEs found in the Piangua genome were slightly different from the TEs reported in *A. brougthonni*, and *A. kagoshimensis*^17,18^. Therefore, it is important to correlate this result with the evolutionary process and transposable element analysis of *A. tuberculosa.* The number of predicted genes was almost identical to those reported in related species, although this number differed to *C. gigas.*

### Genetic diversity of *A. tuberculosa* on the Pacific Coast

Despite some small conservation efforts made by the local communities, the piangua population has suffered a considerable decline in both localities. This phenomenon could explain the large difference between He and Ho (Table 2), where it is possible to evidence a loss of heterozygosity of ~ 71% (Buenaventura = 68.8% and Iscuandé = 72.9%). It could imply a possible loss of genetic diversity^33^ in both piangua populations. The MAF distribution (Fig. S3) was similar in both localities, suggesting that the allele frequency in both Piangua populations was fairly stable. The nucleotide diversity (Table 2) showed that Buenaventura and Iscuandé shared similar levels of genetic variation.

A demographic analysis of the genotypic data suggests that the Piangua population in both localities has steadily increased after two population expansions around one hundred thousand years ago (Supplementary Fig. 7, Table 2). This result is similar to those observed for other bivalve species^34–36^. However, this analysis is not able to capture recent events (within the last 50 years) in which the populations can be experiencing rapid declines. In this case, the decline is supported by the observed loss of heterozygotes and the consequent decrease in genetic diversity in the piangua population in Buenaventura and Iscuandé. This heterozygote deficiency could be supported by the influence of null alleles and the Wahlund effect^37,38^ and high inbreeding coefficients may be related to the constant process of overexploitation of the resource, considering that Piangua species has been overfished for the last 30 years in Colombia^9^. Interpreting the effective population size was challenging because there is no previous information about Piangua Ne. However, in marine species with high fishing pressure, the Ne values might be smaller and the populations suffer a loss of genetic diversity^39^. Our analysis predicted a Ne value of 895 individuals in the combined population, which is lower than that reported for other bivalves^40–42^.

The expression of important survival traits may be reduced due to inbreeding depression^21,43,44^. This process is common in mollusks due to mortality processes in early life stages^45^ and population reduction. A similar result has been reported in the bivalve *Crassostrea gigas*, where the inbreeding depression caused a reduction in survival rate and growth^46^. Moreover, studies on other marine species, such as *Pagrus auratus*^47^, and *Pleuronectes platessa .L*^39^, have also shown that inbreeding resulted in the disappearance of multiple traits. Therefore, recognizing whether the Piangua population is undergoing a process of high inbreeding is important to propose strategies that favor a population expansion process. By increasing the effective population size^21^ it is possible to improve heterozygosity within populations.

### Population structure of piangua populations in two localities of the Colombian Pacific coast

Establishing the population structure in bivalves is highly challenging, due to the high capacity of migrations by the high flow gene. In the case of Piangua, the STRUCTURE analysis did not show a clear genetic differentiation at the population level between Buenaventura and Iscuandé, even though they are geographically distant, approximately 180 km. Similar to other mollusks, the high genetic similarity between both localities may be attributed to the high capacity of dispersion of Piangua at the larva stage. Additionally, STRUCTURE analysis often struggles to detect weak or subtle genetic structures^27^. In contrast, the DAPC cross-validation analysis (Fig. 4B) and pairwise Fst values suggest a subtle genetic structure between two localities, with a possible sub-structure within Buenaventura. This phenomenon could be explained by a very recent overexploitation of this mollusk in both localities. However, the result can also be attributed to temporal chaotic genetic patchiness (CGP)^48^. Thus, this situation would require close and continuous genetic monitoring of the populations in the two localities to determine if human intervention is indeed causing the structuring of a panmictic population.

The low value of F_st_ (0.004) and the admixture observed within samples in both locations imply that there is a high gene flow between the two populations (see Fig. 4C and Fig. S6). This high gene flow is the consequence of frequent migration among individuals across the localities which is driven by the patterns of oceanic gene flow across the Colombian pacific coast^49^ and the capacity of the species to disperse over large distances in the early life stages^50^. However, the p-values among the sampling points in Buenaventura showed a significant difference between points 1 and 3 compared to point 2 (Table S1), a result corroborated by DAPC analysis (Fig. 4B). This finding suggests the development of a recent sub-structure in Buenaventura, likely driven by the loss of heterozygosity or CGP^48^.

The results obtained in this study agree with the analysis of Diringer et al., which showed no differentiation in population structure between two *A. tuberculosa* populations that are also located north of the equator^50^. They analyzed the COI mitochondrial marker in 48 specimens of two localities above the equator, 24 for Esmeralda, Ecuador, and 24 for Tumaco, Colombia^50^. They showed that the F_st_ between these localities was 0.011 and argued that the main reason for this result is that the trochophore larvae can disperse for a few hundred kilometers^50^. Also, some studies have demonstrated that oceanic currents can mark patterns in the genetic variations of mollusks such as *A. broughtonii* and *Pinctada maxima*^37,50–52^.

### Conservation implications

One of the most critical concerns for the community in these regions is the decline of the piangua population over the years. Marine conservation strategies must be improved, and research efforts must catch up. One main reason is the lack of knowledge of marine species^53,54^, mainly in the genetic field. Providing genetic insight allows an understanding of the evolutionary process, population dynamics, and genetic flow, contributing to the design of conservation strategies^54–57^. In this study, we found that piangua populations in two localities of the Colombian Pacific coast have remained stable for several thousand years. However, we observed a recent and considerable loss of genetic diversity, and a very subtle population structure with a probable sub-structure in Buenaventura likely influenced by overfishing.

Piangua has been subjected to constant overexploitation in recent years, which could lead to a reduction of population size and might be the consequence of the inbreeding process and the loss of diversity^39,58^. Recognizing the genetic information makes it possible to establish strategies such as restoration interventions and environmental monitoring^54,59^. Thus, it is crucial to intensify efforts to study the broad dispersal range of Piangua to gain a comprehensive understanding of its current population status. It is essential to establish correlations between this status and the effects of the fishing pressure and its impact on the weak population structure. Additionally, the potential role of private alleles of each locality should be assessed since some of them could be involved in early adaptation processes to each environment^60^.

## Materials and methods

### Sample collection

Specimens were collected from two localities on the Colombian Pacific coast, Buenaventura, in the Valle del Cauca department, and Iscuandé, in the Nariño department (Fig. 1). The collection points were selected based on information provided by local communities. All specimens had an average size of 5 cm in diameter and were not discriminated between females and males. For Buenaventura, 51 specimens were collected from three mangrove swamps: 17 from mangrove 1 (3.846030, −77.283847), 16 from mangrove 2 (3.900195, −77.294537), and 18 from mangrove 3 (3.8747614, −77.2935579). For Iscuandé, 43 specimens were collected from three mangrove swamps: 15 from mangrove 4 (2.61278, −78.01833), 14 from mangrove 5 (2.64306, -78.01806), and 14 from mangrove 6 (2.63889, −78.08917). The specimens were dissected, and a piece of the adductor muscle and mantle of each specimen was extracted and conserved separately in 1 mL of salt-saturated DMSO buffer (20% DMSO, 250 mM EDTA pH 8, and NaCl to saturate the solution)^61^. The remaining tissue was conserved in Falcon tubes with approx. 10 mL of salt saturated DMSO buffer. All specimens were brought to the Mycology and Phytopathology Laboratory (LAMFU), where each specimen’s genomic DNA was extracted.

### Genome sequencing and assembly

Tissues from two specimens collected at Buenaventura were sent to the Vertebrate Genomes Laboratory (VGL) of Rockefeller University, where whole-genome sequencing was done using PacBio-Hifi and Arima technologies. According to their protocols, VGL extracted the genomic DNA (gDNA) for sequencing. The data from PacBio-Hifi was analyzed using NanoPlot^62^. The genome assembly was performed through *de novo* methodology using the Hifiasm assembler^63^ with default parameters, a ploidy of two, and the Hi-C reads integration with the tags --h1 and --h2. The scaffolding was performed through Salsa^64^ according to its indications, and then it was evaluated using Juicertools (https://github.com/aidenlab/juicer*).* The assembly was assessed using Quast^65^ and BUSCO^66^ with the mollusca_odb10 database. The mitochondrial genome was identified through local alignment via blastn command, with our chromosome-level genome as the query and the mitochondrial genome of *Anadara broughtonii* (OM807134.1) as the target. The identified scaffold corresponding to the mitochondrial genome was then isolated and analyzed on the BLAST website (https://blast.ncbi.nlm.nih.gov/*)* against *Anadara* taxon (taxid:6554).

The transposable elements were identified through the EDTA pipeline^67^; the assembly was then masked using the NGSEP GenomeAssemblyMask command. The genes were annotated using MAKER^68^, which received the masked assembly, and the *A. broughtonii* proteome, downloaded from MolluscoDB (http://mgbase.qnlm.ac/home*).* The annotation was evaluated using the NGSEP TranscriptomeAnalyze command, and the synteny was performed through the JCVI MCscan pipeline^69^. Finally, a functional analysis was performed through eggNOG-mapper v2^70^ using as input the *A. tuberculosa* proteome obtained from the annotation evaluation.

### Genomic DNA extraction

The genomic DNA was extracted from each specimen using the CTAB method^71^. Briefly, approx. 30 mg of adductor muscle was incubated with 600µL of CTAB buffer (100 mM Tris-HCl pH 8, 1.4 M NaCl, 20 mM EDTA, and 2% CTAB) supplemented with 0.2% Mercaptoethanol and 0.1 mg/mL proteinase K (New England Biolabs) at 60 °C for 1 h, then the genomic DNA was extracted using 500µL of chloroform: isoamyl alcohol (24:1). The mix was centrifuged at 10,000 x g for 5 min, the supernatant was collected in a new tube, and 300µL of isopropanol were added and incubated at −20 °C overnight. The DNA was harvested by centrifugation at 10,000 x g for 5 min and then washed once with 1 mL 70% of ice-cold ethanol. The pellet was air-dried at room temperature. Finally, the DNA was resuspended in 70µL of DNase and RNase-free water. The RNA contamination was eliminated through digestion using 1 mg/mL RNAse A (New England Biolabs) at 37 °C for 1 h. The concentration was estimated using Qubit™ 4 (Thermo Scientific) by the Core Facility - GenCore (Universidad de Los Andes), and the quality was evaluated on a 1% agarose gel.

### Genotyping analysis

A total of 89 specimens of piangua were genotyped through DArTseq technology^11^. Approximately 50 to 100 ng of genomic DNA for each individual was used for this analysis. The DArT libraries were generated by the digestion of genomic DNA with *Pst*I and *Taq*I restriction enzymes, then the fragments were ligated with Illumina adaptors and sequenced on Illumina (Illumina Technologies). DArT sequencing data were obtained for 49 Buenaventura and 40 Iscuandé specimens. The barcode sequences were removed from the raw reads using Trimmomatics^72^, and the 3’-end was cleaned up from the adaptor sequence using an awk command. The raw reads with sizes greater than 40 bases were kept and stored in a new fastq file.

SNP discovery and genotyping were performed following the reference-guided pipeline implemented in NGSEP v 4.3.1^73^. The raw reads were aligned to the piangua genome using the NGSEP ReadsAligner command. Then, the bam files were sorted by Picard software (https://github.com/broadinstitute/picard). The SNPs were detected using the NGSEP MultisampleVariantsDetector command with the sorted bam files and the tag -minMQ with a value of 30. Other parameters were used by default.

The dataset of genotyped SNPs was stored in variant call format, which is the standard for storing DNA polymorphism data^74^. The VCF was filtered to obtain only bi-allelic SNPs and SNPs with a minor allele frequency greater than 0.01. The NGSEP VCFFilter command was used with tags -s, -minMAF with a value of 0.01, -q of 40, and -m with a value of 50 to retain only SNPs genotyped in at least 50 individuals. Multiallelic SNVs were not taken into account because they were only 592 (10.17% of the total), and the allele frequency of the third allele was on average lower than 0.04. Diversity statistics were obtained from the filtered VCF using the NGSEP commands VCFSummaryStats and VCFDiversityStats. Also, for each locality, a VCF file was obtained from the filtered VCF using the NGSEP VCFFilter command and the -saf tag, which receives a file with the IDs of the samples for each locality. These VCFs kept the bi-allelic SNPs, and the MAF filter was not applied to keep the private allele for each locality.

### Population genetics and clustering

The SNPs were analyzed for the Hardy-Weinberg Equilibrium with the test proposed by Wigginton et al.,^75^ implemented in the vcftools package^74^. The raw p-values were corrected for multiple testing using the Bonferroni method. So, the expected heterozygosis (H_e_), observed heterozygosis (H_o_), and the Weir and Cockerham^76^ fixation indexes F_st_, F_is,_ and F_it_ were determined using the Adegenet^77^, the Hierfstat^78^, and Pegas packages. The p-values for Fst pairwise were estimated using the gl.fst.pop function of the dartR package. For this, we implemented a bootstrap of 10000 and a confidence interval of 95%. The nucleotide diversity (π) was estimated using vcftools with a window of 10,000,000 bp, and the Tajima’ D index was calculated with the SAMBAR_v1.10 package^79,80^. The discriminant analysis principal component (DAPC) was carried out using the DAPC cross-validation function from Adegenet R packages where 30 principal components (PCs) were retained to achieve the lowest mean squared error (MSE), and the plot was performed using the scatter.dapc function. The gene flow analysis was estimated using the divMigrate function of diveRsity R packages^81^. All plots were performed using the ggplot2 package^82^.

The population structure analysis between localities was analyzed using STRUCTURE with the admixture model^83^. The VCF file was transformed into a STRUCTURE format by the NGSEP VCFConverter command with the -structure tag. In the mainparams file, the burn-in parameter was modified at 20,000 and the numreps parameter at 50,000 sampling iterations. Finally, STRUCTURE was run to determine the optimal number of populations or genetic clusters (K) with K values ranging from 1 to 6, with 20 replicates for each K. The best K was estimated using the Evanno et al. method^84^, and the snmf function of the LEA R package^85^. Additionally, population structure was estimated using the superparamagnetic clustering method which was integrated into the NetView R package^86^.

Finally, the effective population size (Ne) was estimated using the linkage disequilibrium model (LD) implemented in NeEstimator^87^. Ne was analyzed for the entire population (combining Buenaventura and Iscuandé), as well as for each locality. Additionally, the population history was inferred using the extended Bayesian skyline plot, following the tutorial from Trucchi et al., (2014)^88^. Briefly, the VCF file was converted to NEXUS format using the script vcf2phylip.py^89^. The extended Bayesian skyline plot was performed through BEAST2^90^ using a substitution rate of 1.0, the clock rate of 1.0, MCMC chain length of 1,000,000, and the remaining parameters by default. Finally, the plot was done using ggplot2.

## Electronic supplementary material

Below is the link to the electronic supplementary material.

Supplementary Material 1

Supplementary Material 2

## Data Availability

The genome of this article is available in NCBI with the BioProject ID PRJNA997345.
