# Supplementary material for: Genetic diversity of Anadara tuberculosa in two localities of the Colombian Pacific Coast
Source: Sci Rep. 2024 Nov 18;14:28467. doi: 10.1038/s41598-024-78869-3 (PMC11574214; doi:10.1038/s41598-024-78869-3)
Supplement: Supplementary file 1 — Supplementary Material 1 [file 41598_2024_78869_MOESM1_ESM.docx]

**Supplementary Table 1. Pairwise F_st_ index between each mangrove.**

|  |  | **Buenaventura** | | | **Iscuandé** | | |
| --- | --- | --- | --- | --- | --- | --- | --- |
|  | **Mangrove** | **1** | **2** | **3** | **4** | **5** | **6** |
| **Buenaventura** | **1** | 0 | - | - | - | - | - |
|  | **2** | 0.0085 | 0 | - | - | - | - |
|  | **3** | 0.0015 | 0.0102 | 0 | - | - | - |
| **Iscuandé** | **4** | 0.0059 | 0.0041 | 0.0101 | 0 | - | - |
|  | **5** | 0.0036 | 0.0075 | 0.0090 | -0.0033 | 0 | - |
|  | **6** | 0.0094 | 0.0059 | 0.0062 | -0.0022 | -0.0022 | 0 |

Symmetric matrix of pairwise F_st_ value between each mangrove. The F_st_ was estimated according to the method of Weir and Cockerham ^64^. Red values are significantly higher than zero.

**Supplementary Table 2.** Estimated effective population size for the entire Piangua population (combined Buenaventura and Iscuandé samples), Buenaventura samples, and Iscuandé samples**.**

| **Population** | **samples** | **LD method** |
| --- | --- | --- |
|  |  | **95% CI (lower**  **uper)** |
| **Entire** | 89 | 537 |
|  |  | 1253 |
| **Buenaventura** | 49 | 538.4 |
|  |  | Infinite |
| **Iscuandé** | 40 | 709.2 |
|  |  | Infinite |

Ne was estimated using the LD method implemented by NeEstimator v2
