# Supplementary material for: Genetic diversity of Anadara tuberculosa in two localities of the Colombian Pacific Coast
Source: Sci Rep. 2024 Nov 18;14:28467. doi: 10.1038/s41598-024-78869-3 (PMC11574214; doi:10.1038/s41598-024-78869-3)
Supplement: Supplementary file 2 — Supplementary Material 2 [file 41598_2024_78869_MOESM2_ESM.docx]

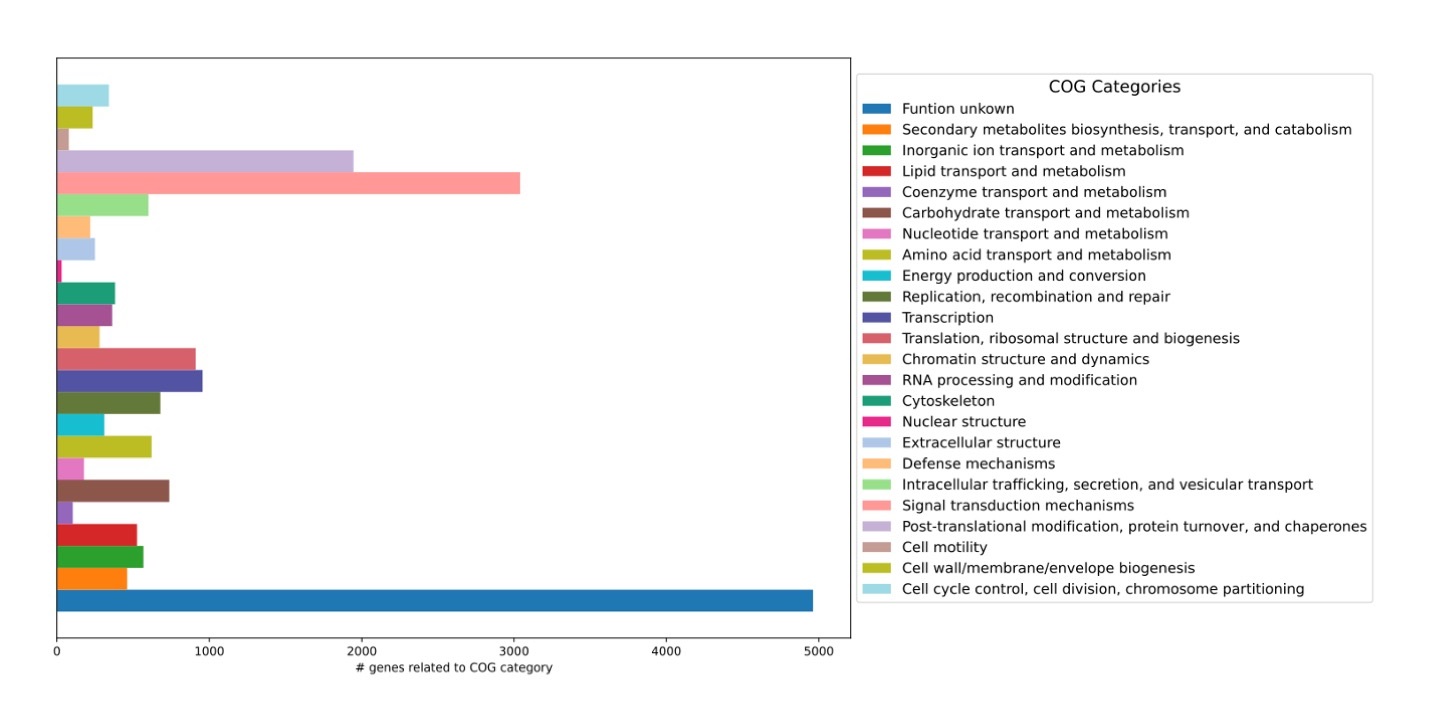


**Supplementary Figure 1. Functional analysis.** This graph represents the distribution of the COG categories for the predicted proteins. The y-axis represents the COGs, which in turn are represented by a color, and the x-axis represents the number of proteins in these COGs.

**
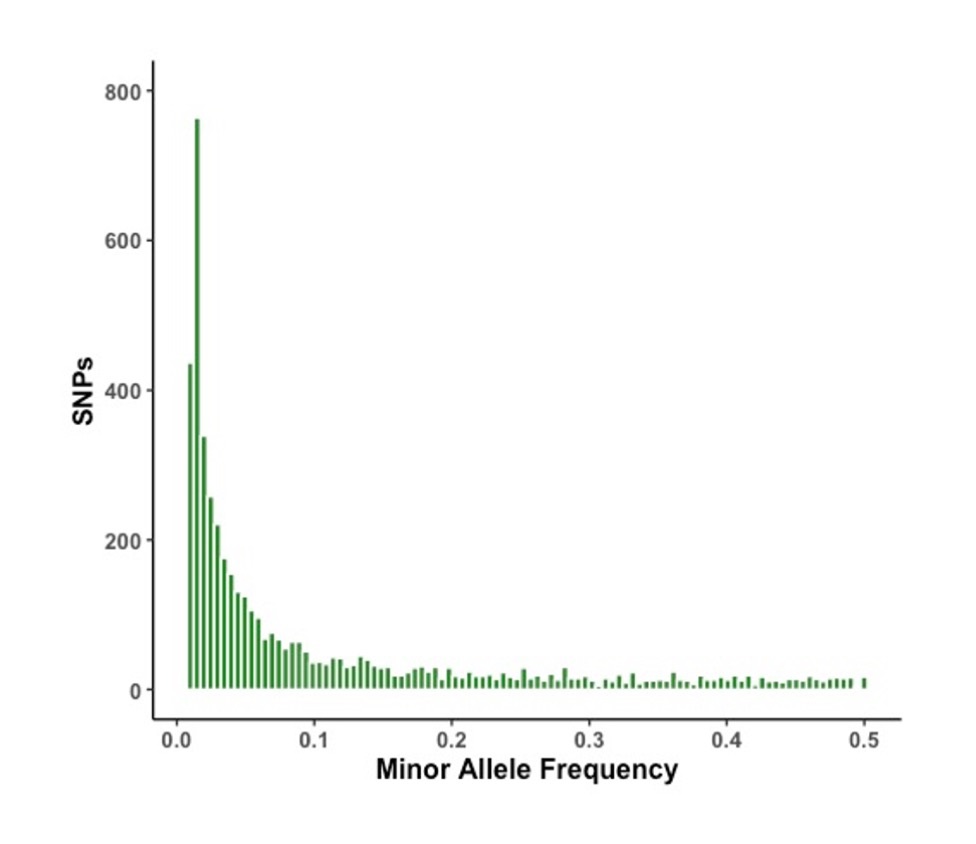
**

**Supplementary Figure 2. Distribution of Minor Allele Frequency (MAF).** The green bars represent the MAF distribution of 4,825 SNPs without a locality filter.


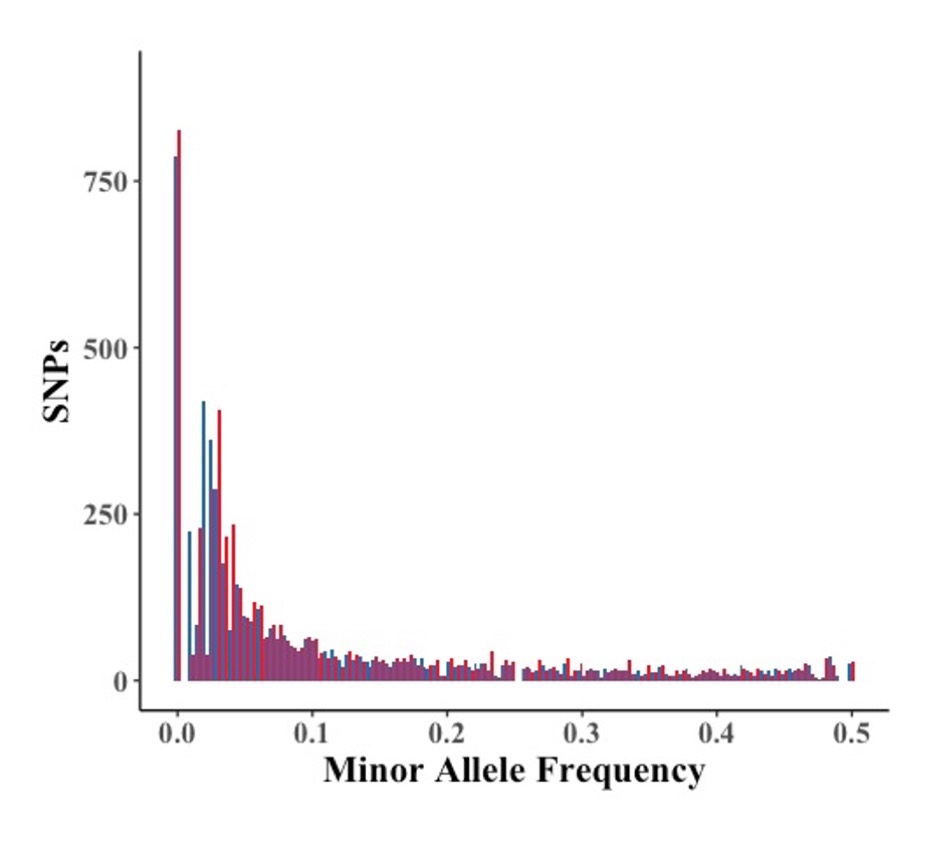


**Supplementary Figure 3. Distribution of the Minor Allele Frequency (MAF) in both localities.** The blue bars are the MAF for Buenaventura, and the red bars are for Iscuandé.


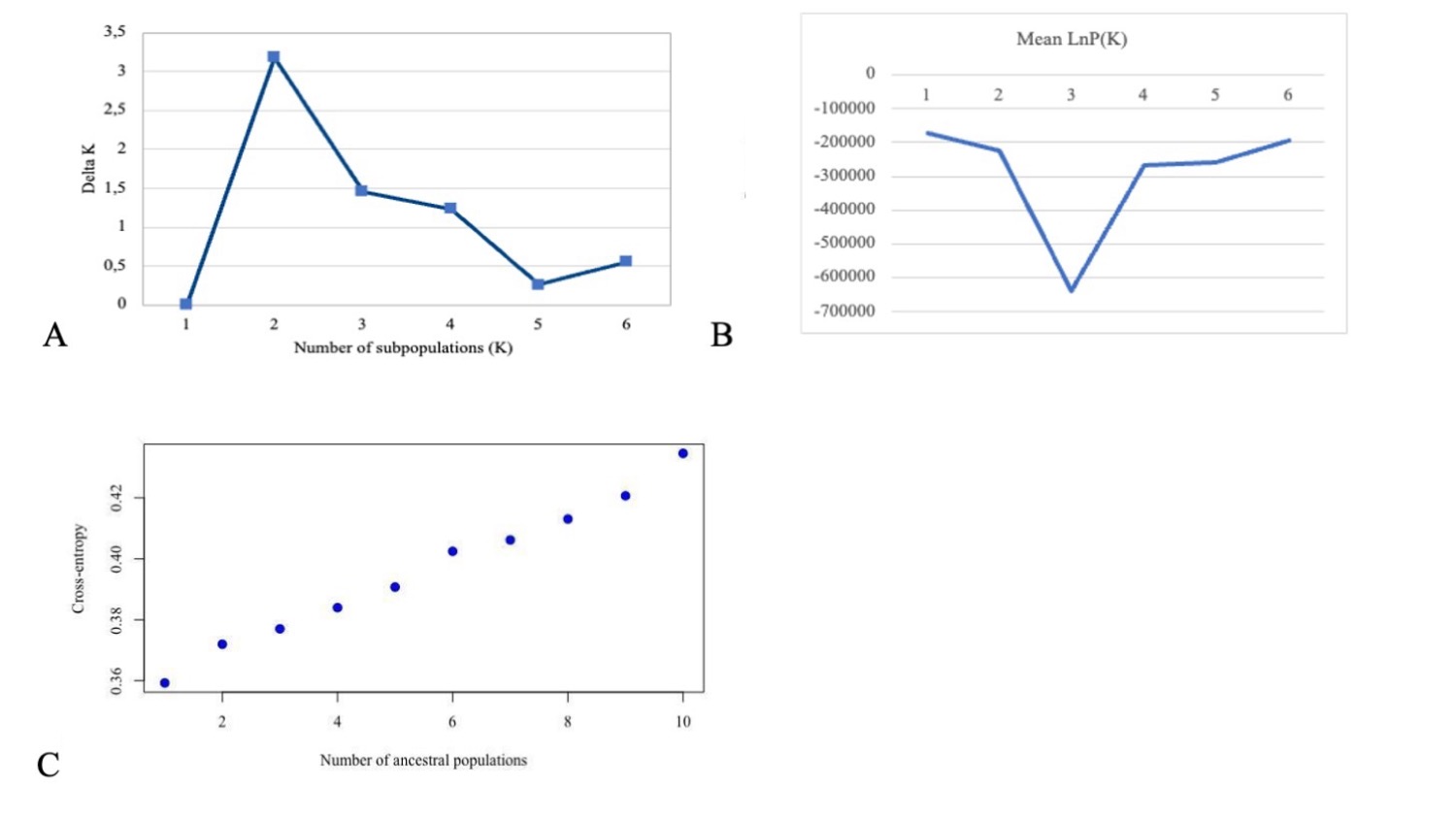


**Supplementary Figure 4.** Estimation of the number of populations. **A**. The method of Evanno et al^21^. The peak at K two represents the best number of subpopulations, **B**. mean likelihood probability (LnP(K)), the y-axis is the mean log likelihood probability, and the x-axis is the K clusters. C. The snmf function of the LEA R packages, the best number of ancestral population was one.


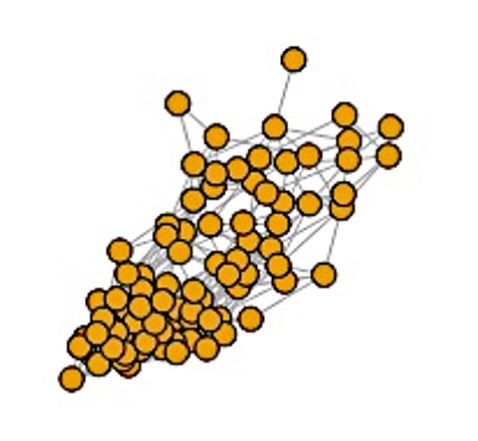


**Supplementary Figure 5.** Estimation of population structure using the unsupervised network clustering method. The estimation was performed using NetView R packages. The yellow nodes represent individuals and the gray lines represent the relationship between individuals (edge).


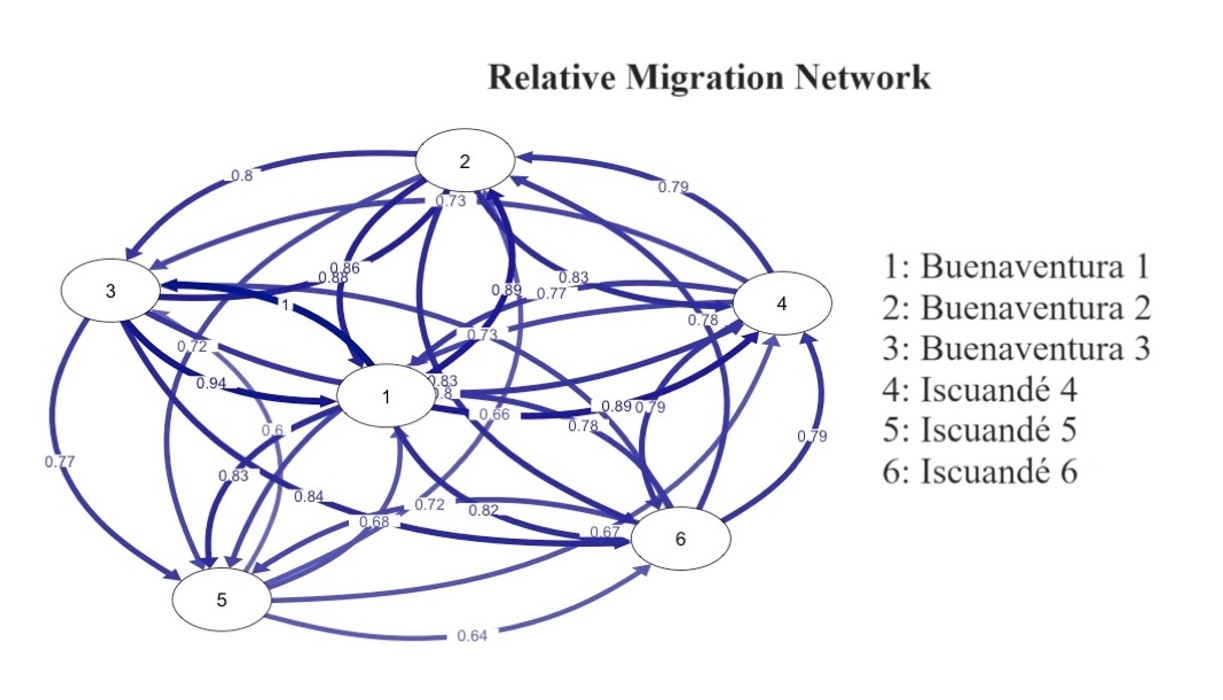


**Supplementary Figure 6.** Guided graph representing gene flow between Buenaventura and Iscuandé sampling points. Blue arrows indicate genetic migration between each sampling point.


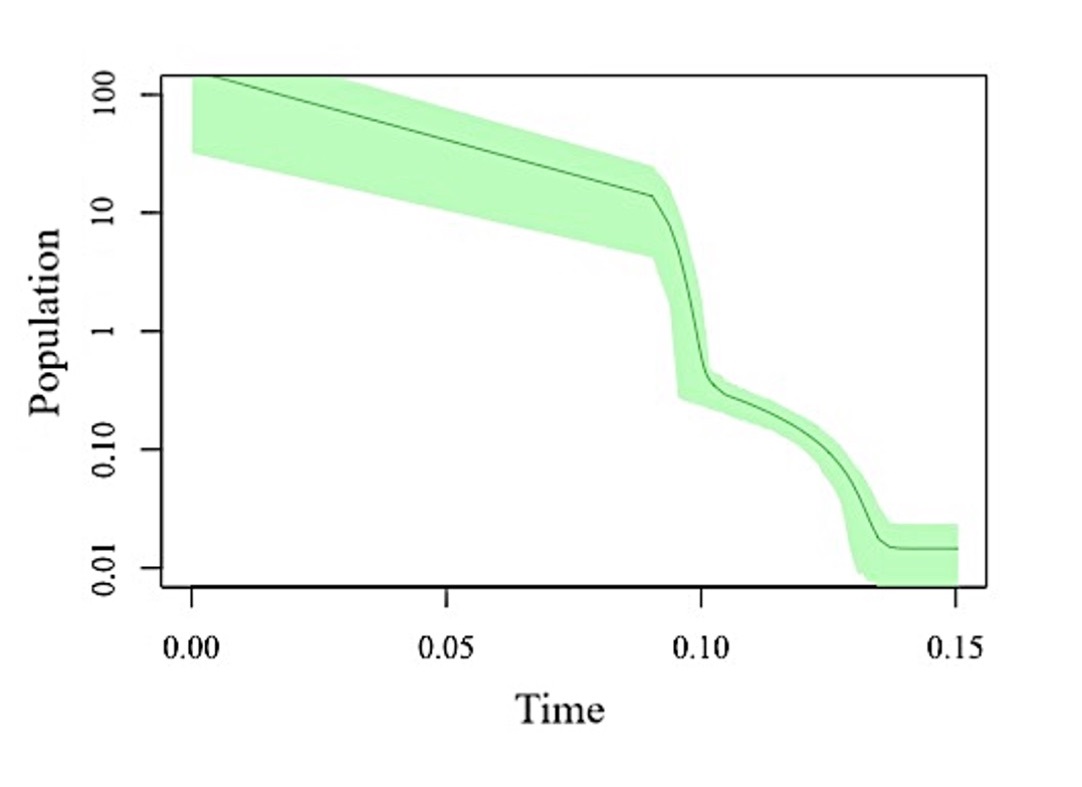


**Supplementary Figure 7. Extended Bayesian Skyline plot.** The EBSP represents the evolutionary trajectory of the effective population size of Piangua at the two locations over time. The central trend of the population size, represented in a black line, is complemented by the lower and upper bounds encapsulating its 95% Maximum Posterior Density (HPD) (green region). The Y-axis is the effective size (Ne) and the X-axis is the time in millions of years (My).


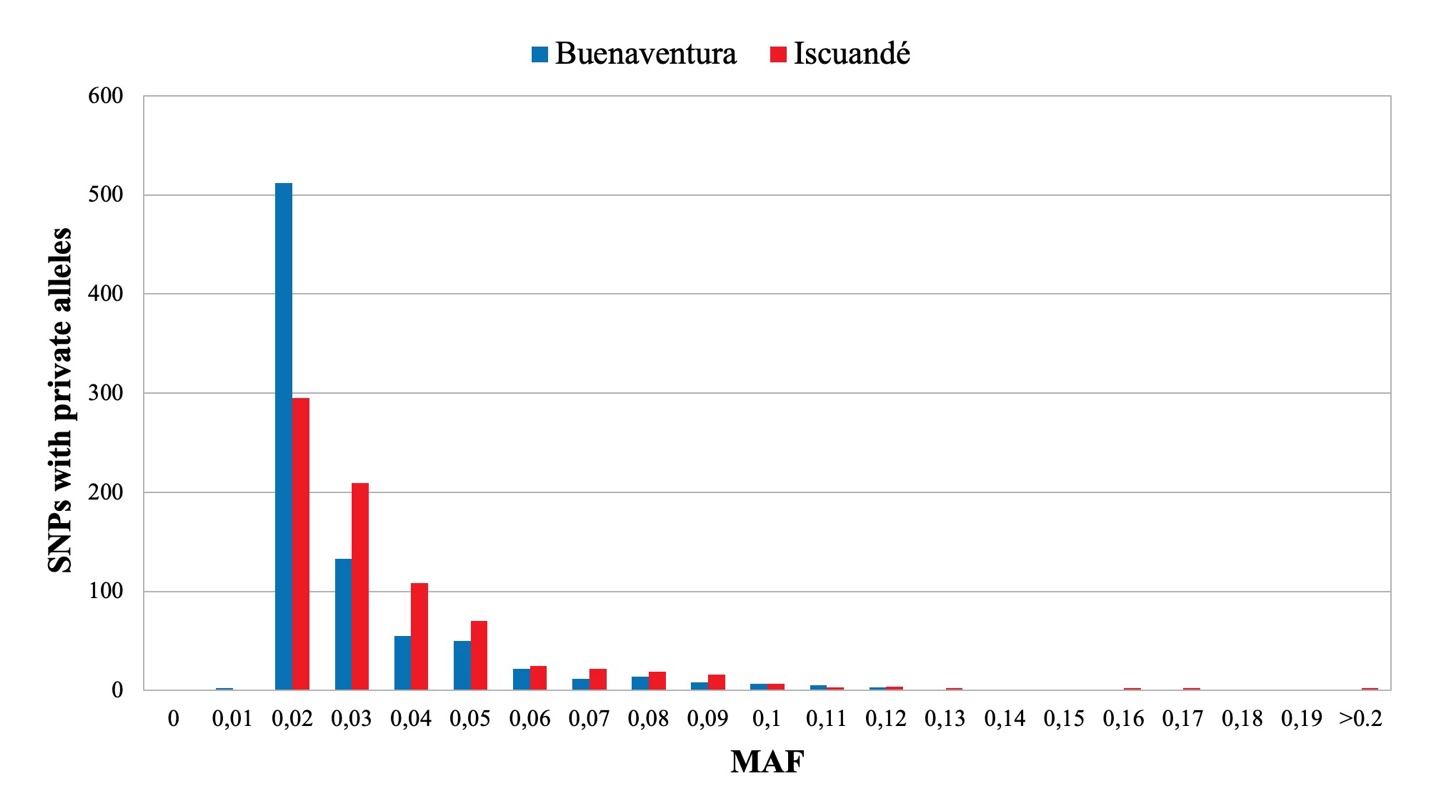


**Supplementary Figure 8. Distribution of the Minor Allele frequency for private alleles in both localities.** The blue bars are the MAF for Buenaventura, and the red bars are for Iscuandé.
